# Feasibility study of *Syngo* iFlow in predicting hemodynamic improvement post-endovascular procedure in peripheral artery disease

**DOI:** 10.1186/s12872-024-03762-w

**Published:** 2024-02-10

**Authors:** Ming Tang, Fanyi Zeng, Xindong Chang, Mingfei He, Qingqing Fang, Lele Xue, Xinyi Luo, Shiwu Yin

**Affiliations:** 1 Department of Interventional Vascular Medicine, Hefei Hospital Affiliated to Anhui Medical University, The Second People’s Hospital of Hefei, 574 Changjiang East Road, Yaohai District, Hefei City, 230011 Anhui Province China; 2 The Fifth Clinical College of Medicine, Anhui Medical University, 1166 Wangjiang West Road, Shushan District, Hefei City, 230011 Anhui Province China

**Keywords:** Peripheral arterial disease, Endovascular procedure, *Syngo* iFlow, Perfusion angiography: percutaneous transluminal angioplasty

## Abstract

**Objective:**

This study endeavors to examine the feasibility of predicting the clinical outcomes of patients suffering from peripheral artery disease (PAD) who undergo endovascular intervention, by employing the *Syngo* iFlow technology.

**Methods:**

Retrospectively enrolling 76 patients from December 2021 to May 2023, yielding a total of 77 affected limbs, this study employs clinical outcomes (improvement or otherwise) as the gold standard. Two physicians conducted visual assessments on both DSA and iFlow images to gauge patient improvement and assessed inter-observer consistency for each image modality. The Time to Peak (TTP) of regions of interest (ROI) at the femoral head, knee joint, and ankle joint was measured. Differences in pre- and post-procedure TTP were juxtaposed, and statistically significant parameter cutoff values were identified via ROC analysis. Employing these cutoffs for TTP classification, multivariate logistic regression and the C-statistic were utilized to assess the predictive value of distinct parameters for clinical success.

**Results:**

Endovascular procedure exhibited technical and clinical success rates of 82.58 and 75.32%, respectively. Diagnostic performance of iFlow image visual assessment surpassed that of DSA images. Inter-observer agreement for iFlow and DSA image evaluations was equivalent (κ = 0.48 vs 0.50). Post-classification using cutoff values, multivariate logistic regression demonstrated the statistical significance of ankle joint TTP in post-procedure iFlow images of the endovascular procedure for clinical success evaluation (OR 7.21; 95% *CI* 1.68, 35.21; *P* = 0.010), with a C-statistic of 0.612.

**Conclusion:**

*Syngo* iFlow color-encoded imagery holds practical value in assessing the technical success of post-endovascular procedures, offering comprehensive lower limb arterial perfusion visualization. Its quantifiable parameters exhibit promising potential for prognosticating clinical success.

## Introduction

Peripheral Artery Disease (PAD) is a syndrome of impaired peripheral circulation caused by narrowing or obstruction of lower limb arteries, resulting in reduced blood flow [1]. PAD has various clinical manifestations. According to guidelines established by the American College of Cardiology (ACC) and the American Heart Association (AHA), PAD is calssified into four primary classifications based on its symptoms and signs: asymptomatic, intermittent claudication, chronic limb ischemia, and acute limb ischemia [2]. Among these, lower limb ischemia represents the most severe clinical manifestation, with rest pain, intermittent claudication, and ischemic ulcers as typical symptoms. Without timely intervention, these symptoms may progress irreversibly, necessitating amputation. Statistically, the amputation rate can be as high as 20%, while patients with PAD face a staggering 50% mortality within 5 years [3]. When lower limb ischemic symptoms manifest, prompt evaluation and proactive implementation of revascularization measures are imperative to reduce amputation risk. Presently, endovascular procedures stands as the preferred modality for vascular revascularization. During the course of endovascular procedures, Digital Subtraction Angiography (DSA) is employed to visualize the location and extent of stenotic segments, concurrently assessing the vascular reconstruction. In addition to DSA, several studies have explored alternative methods to assess the effectiveness of vascular reconstruction. These include the Ankle-Brachial Index (ABI), Transcutaneous Oxygen Pressure Measurement (TcPO2), and Near-Infrared Spectroscopy (NIRS). While ABI provides a convenient and reproducible approach for evaluating lower limb hemodynamics, it is susceptible to the influence of small arteries and arterial calcification [4]. TcPO2, belonging to metabolic measurements, is insensitive to the degree of arterial hardening and can predict ulcer healing or reduce amputation range by assessing tissue healing probability [5]. NIRS, a relatively new technology garnering attention in peripheral vascular disease, calculates oxygen saturation and perfusion changes in superficial tissues through spectral analysis, offering quantifiable data on oxygen saturation and perfusion changes in the subcutaneous superficial muscles [6]. However, both NIRS and TcPO2 are influenced by skin conditions and fat thickness, limiting their accuracy. Concurrently, imaging technologies, integral to clinical diagnosis, play a crucial role. Techniques such as Duplex Ultrasound (DUS), Computed Tomography Angiography (CTA), and Non-Contrast Magnetic Resonance Angiography have proven effective in diagnosing lower limb vascular stenosis [7, 8]. However, these techniques lack intraoperative feasibility, requiring pre- or post-operative application. As the gold standard for diagnosing vascular diseases, DSA holds particular importance during endovascular procedures. Operators assess arterial perfusion preliminarily through contrast bolus chasing angiography. Presently, DSA image interpretation relies on subjective judgment, making it challenging to establish quantifiable standards.

The *Syngo* iFlow technology developed by Siemens holds promise in addressing the aforementioned issues. This innovative approach converts conventional grayscale DSA images into color-coded images. By assigning distinct colors based on the contrast agent’s concentration variations, this technique depicts the temporal distribution of the contrast agent within vessels. Additionally, it enables the creation of time-intensity curve (TIC) graphs for regions of interest (ROI), thereby providing quantitative blood flow data. Previous research indicates the potential advantages of this technology in assessing intravascular therapeutic efficacy. In line with Li et al.’s [9] research, iFlow assessment following interventional treatment for acute lower limb arterial ischemia revealed significant reductions in postoperative time to peak (TTP) at both the knee and ankle regions, compared to preoperative measurements. Furthermore, postoperative ankle TTP was markedly higher in amputees compared to patients with complete or partial relief post-intervention. And the TTP difference between pre- and post-intervention in the ankle and knee regions showed a strong correlation with ABI and TcPO2 differences. These findings elucidate that the iFlow parameters not only exhibit utility in the evaluation of PAD therapeutic efficacy but also offer quantifiable parameters, thereby lending support to further analyses. Building upon the aforementioned context, this research aims to assess iFlow color-coded imagery and its quantitative parameters acquired during the endovascular procedure, thereby exploring their feasibility in predicting clinical outcomes. This research holds promise in introducing novel perspectives and methodologies for postoperative prognostication of therapeutic efficacy.

## Methods

### Patients

This research employed a retrospective research design, enrolling a cohort of 76 PAD patients admitted to our institution between December 2021 and May 2023 as the subjects of investigation. The inclusion criteria were as follows: (1) All chronic lower limb ischemia patients strictly met the diagnostic criteria for PAD [10]; (2) Symptoms persisted despite conservative treatments such as medication and exercise therapy; (3) Preoperative imaging confirmed > 50% stenosis in above-the-knee arteries and unclear below-the-knee angiographic visualization; (4) Preferred treatment is the endovascular procedure. The exclusion criteria were: (1) Complete occlusion of below-the-knee arteries; endovascular angioplasty unsuccessful; (2) Incomplete or missing clinical data; (3) Inconsistent angiographic catheter placement before and after imaging; (4) Poor iFlow imaging quality due to limb movement during imaging; (5) Patients with severe cardiovascular or cerebrovascular diseases, or other congenital conditions.

### Surgical procedure and post-processing of iFlow

**Fig. 1:**
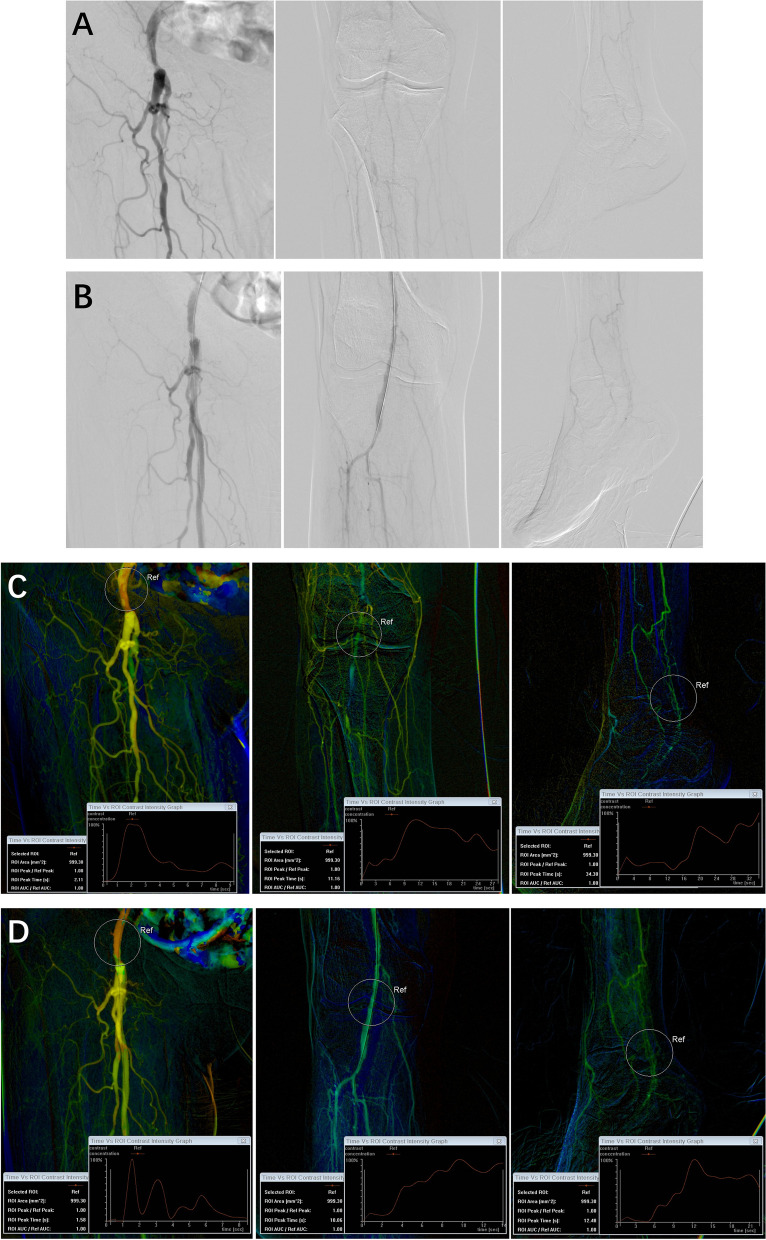
DSA and iFlow images of an 83-year-old female patient, who underwent right femoral superficial artery stent placement, anterior tibial, posterior tibial, and peroneal artery balloon angioplasty. **A, C** pre-endovascular procedure; **B, D**) post-endovascular procedure, showing significant hemodynamic improvement

**Fig. 2:**
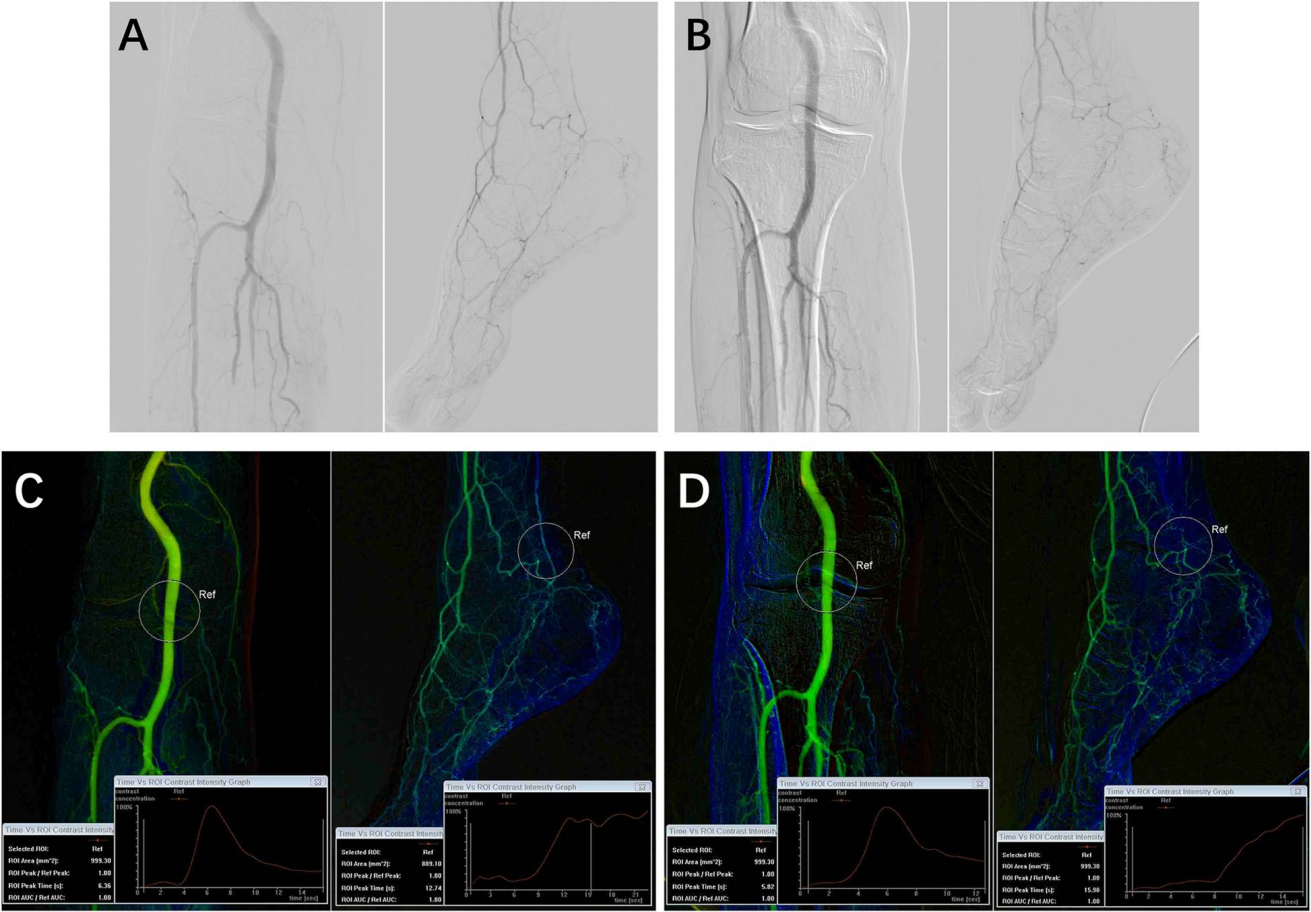
DSA and iFlow images of a 74-year-old male patient, who underwent balloon angioplasty of the posterior tibial and peroneal arteries. **A, C** pre-endovascular procedure; **B, D**) post-endovascular procedure, showing no improvement in hemodynamics

Under local anesthesia, percutaneous access was achieved through the femoral artery or femoral superficial artery using a 5F angiographic catheter (Cordis). The procedure was conducted using the Siemens ARTIS pheno imaging system (Siemens Healthcare GmbH, Forchheim, Germany), with contrast administration facilitated by a high-pressure injector (Bayer Mark 7 Arterion, USA) delivering iohexol (32 g/ml). The angiographic catheter tip was positioned at the hip joint space for staged comprehensive lower limb angiography: The upper limit of Stage 1 encompassed the catheter tip and femoral head, providing clear visualization of the femoral superficial and deep arteries; Stage 2’s lower boundary encompassed the entire knee joint; Stage 3’s upper limit was situated 5 cm above the patella’s superior edge; Stage 4 included the entire foot. Contrast agent volume for the initial three stages were 9 ml each, and 15 ml for Stage 4; injection pressure was maintained at 300 PSI, with a contrast delivery rate of 3 ml/s, and an imaging frame rate of 15 frames/second. Following manual contrast bolus chasing angiography, balloon dilation is employed to dilate narrowed arteries. Stent insertion follows if unsatisfactory results or failure occurs (e.g., residual stenosis > 50%, the development of flow-impairing dissection, or persistent pressure difference) [11]. Postoperatively, the corresponding stenotic sites were reassessed using identical protocols. Using the Siemens workstation, color-coded images were reconstructed, and 1000mm^2^ ROIs were chosen in the femoral head, knee joint, and ankle joint areas for TTP measurement. Within the femoral head ROI, the center was positioned above the bifurcation of the common femoral artery (ROI 1). For the knee joint ROI, the center was located at the popliteal artery of the knee joint space (ROI 2). In the ankle joint ROI, the center was situated at the targeted vessel location within the ankle joint space (ROI 3). The TTP measurements in these specified locations were repeated thrice to attain an average value (Figs. 1 and 2).

### Definition of clinical outcomes

Post endovascular procedure, the outcome is defined in terms of technical success and clinical success. Technical success is characterized by a residual stenosis of < 30% above the popliteal lesion and successful revascularization of the target vessel through balloon angioplasty [12]. On the other hand, Clinical success is defined as disappearance or partial relief of intermittent claudication, rest pain, and relevant symptoms, as documented in the patient’s electronic records within 3–7 days postoperatively (with Rutherford classification decreasing by at least one level, e.g., from IV to III), and a decrease of at least one level in ABI (e.g., from severe to moderate). Additionally, it includes ulcer healing, cancellation of planned amputation, or obviation of the need for amputation after flap surgery.

### Image analysis

Two experienced interventional vascular physicians (A and B), blinded to surgical processes and outcomes, conducted visual assessments. Comparative analyses of pre- and post-endovascular procedure DSA images were performed. The evaluators independently assessed both the DSA angiographic images and the color-coded images reconstructed using iFlow. A 4-point scoring system was employed for evaluation: 0, worse; 1, no change; 2, improved < 50%; 3, markedly improved ≥50%. Based on this criterion, scores of 0 and 1 were considered a negative decision regarding blood flow or perfusion, while scores of 2 and 3 were deemed positive.

### Statistical analysis

Statistical analyses utilized R studio software (v4.2.3, http://www.rproject.org/). The Shapiro-Wilk test assessed normality for continuous data. Normally distributed metric data were presented as mean ± standard deviation ($\overline{x}$*± s*), and paired t-tests were used for between-group comparisons. Non-normally distributed metric data were expressed as *M* (*Q1*, *Q2*), and between-group comparisons employed the Mann-Whitney *U* test. Count data were presented as frequencies (%), with between-group comparisons conducted using the *χ*^*2*^ test or Fisher’s exact test when appropriate.

Using clinical success as the gold standard, two physicians (A and B) assessed the accuracy of vascular reconstruction efficacy evaluations in both DSA and iFlow images. Interobserver agreement for visual scoring results in DSA and iFlow images was analyzed by the two physicians, with negative decisions (indicating improved blood flow or perfusion) scored as 0 or 1, and positive decisions scored as 2 or 3. The Kappa test for evaluation Interobserver agreement, with the coefficient interpreted as follows: slight (0.00–0.20), fair (0.21–0.40), moderate (0.41–0.60), substantial (0.61–0.80), almost perfect (0.80–1.00).

Performing Pearson correlation analysis on the statistically different postoperative and preoperative differences in Time to Peak (△TTP) and preoperative to postoperative differences in Ankle-Brachial Index (△ABI) to assess their correlation. Concurrently, conducting Receiver Operating Characteristic (ROC) curve analysis on pre- and post-TTP to determine cutoff values predicting clinical success. Finally, conducting multivariate logistic regression analysis on preoperative and postoperative iFlow quantitative parameters of the 3 ROIs (classified based on the cutoff values), computing their respective C statistics to evaluate the predictive value for clinical success. A significance level of *P* < 0.05 was considered statistically significant.

## Results

### General characteristics and postoperative vascular reconstruction outcomes

The study comprised 76 patients (52 males, 24 females) aged 51–93 years, with an average age of 73.14 ± 12.31 years. Patient characteristics are outlined in Table 1. A total of 264 lesions in 77 limbs were treated, resulting in a technical success rate of 82.58% (218/264). Technical success rates for each lesion were: common iliac artery, 100% (4/4); external iliac artery, 92.86% (13/14); common femoral artery, 88.89% (8/9); superficial femoral artery, 83.08% (54/65); popliteal artery, 77.78% (35/45); tibio-peroneal trunk, 78.26% (18/23); anterior tibial artery, 86.67% (26/30); posterior tibial artery, 87.10% (27/31); peroneal artery, 77.50% (31/40); dorsalis pedis and plantar arteries, 66.67% (2/3).

**Table 1:** Clinical characteristics of 76 patients

Characteristic	%/$\overline{x}\pm s$	*t*	*P*
**Age (year)**	73.14 ± 12.31		
**Sex (male)**	52 (68.42%)		
**Hypertension**	51 (67.11%)		
**Diabetes**	39 (51.32%)		
**Rutherford classification**			
II	2 (2.63%)		
III	23 (30.26%)		
IV	26 (34.21%)		
V	16 (21.05%)		
VI	9 (11.84%)		
**ABI**		14.303	**< 0.001**
preoperative	0.46 ± 0.11		
postoperative	0.79 ± 0.16		
**TTP of ROI 1**		0.495	0.622
preoperative	3.94 ± 0.83		
postoperative	3.86 ± 1.12		
**TTP of ROI 2**		6.223	**< 0.001**
preoperative	12.86 ± 2.36		
postoperative	10.33 ± 2.59		
**TTP of ROI 3**		7.043	**< 0.001**
preoperative	20.30 ± 2.76		
postoperative	16.38 ± 3.56		

The clinical success rate was 75.32% (58/77). Clinical success rates for each indication were: intermittent claudication, 100% (17/17); rest pain, 72.22% (13/18); ulcers, 63.33% (19/30); and lower limb artery embolism, 83.33% (10/12). Eight patients required reintervention due to recurrence. Additionally, 10 patients underwent post-endovascular procedure amputation: toe amputation (*n* = 8), below-knee amputation (*n* = 1), and femoral amputation (*n* = 1). Three patients died post-endovascular procedure: acute cerebral infarction (*n* = 1), septic shock (*n* = 1), and acute myocardial infarction (*n* = 1).

### Diagnostic performance of clinical outcome evaluation

Physicians A and B visually assessed iFlow perfusion images (Table 2), showing superior results compared to DSA images in sensitivity, specificity, positive predictive value (PPV), negative predictive value (NPV), and accuracy (Table 3). Inter-rater agreement for the 4-point visual assessment using DSA images by the two physicians was comparable to that using iFlow images (κ = 0.50 vs 0.48), both indicating moderate agreement.

**Table 2:** Clinical outcome assessment and imaging visual scores post percutaneous transluminal angioplasty

Modality	Reviewer	Clinical Outcome	Visual Scores			
			0	1	2	3
DSA	A	Improved	0	13	34	10
		Not improved	2	10	7	0
	B	Improved	0	10	33	14
		Not improved	1	10	8	0
iFlow	A	Improved	0	11	27	19
		Not improved	3	10	5	1
	B	Improved	0	6	30	21
		Not improved	1	12	5	1

**Table 3:** Diagnostic performance of clinical outcome assessment after percutaneous transluminal angioplasty

Modality	Reviewer	Sensitivity (95% *CI*)	Specificity (95% *CI*)	PPV (95% *CI*)	NPV (95% *CI*)	Accuracy (95% *CI*)
DSA	A	41.7(22.1, 63.4)	82.7(69.7, 91.8)	52.6(28.9, 75.6)	75.4(62.2, 85.9)	69.7(58.1, 79.8)
	B	47.4(24.4, 71.1)	82.5(70.1, 91.3)	47.4(24.4, 71.1)	82.5(70.1, 90.0)	73.7(62.3, 83.1)
iFlow	A	50.0(29.1, 70.9)	86.5(74.2, 94.4)	63.2(38.4, 83.7)	78.9(66.1, 88.6)	75.0(63.7, 84.2)
	B	61.1(35.7, 82.7)	86.2(74.6, 93.9)	57.9(33.5, 79.7)	87.7(76.3, 94.9)	80.3(69.5, 88.5)

*CI* Confidence interval, *PPV* Positive predictive values, *NPV* Negative predictive values

### Quantitative analysis of iFlow parameters before and after surgery

**Fig. 3:**
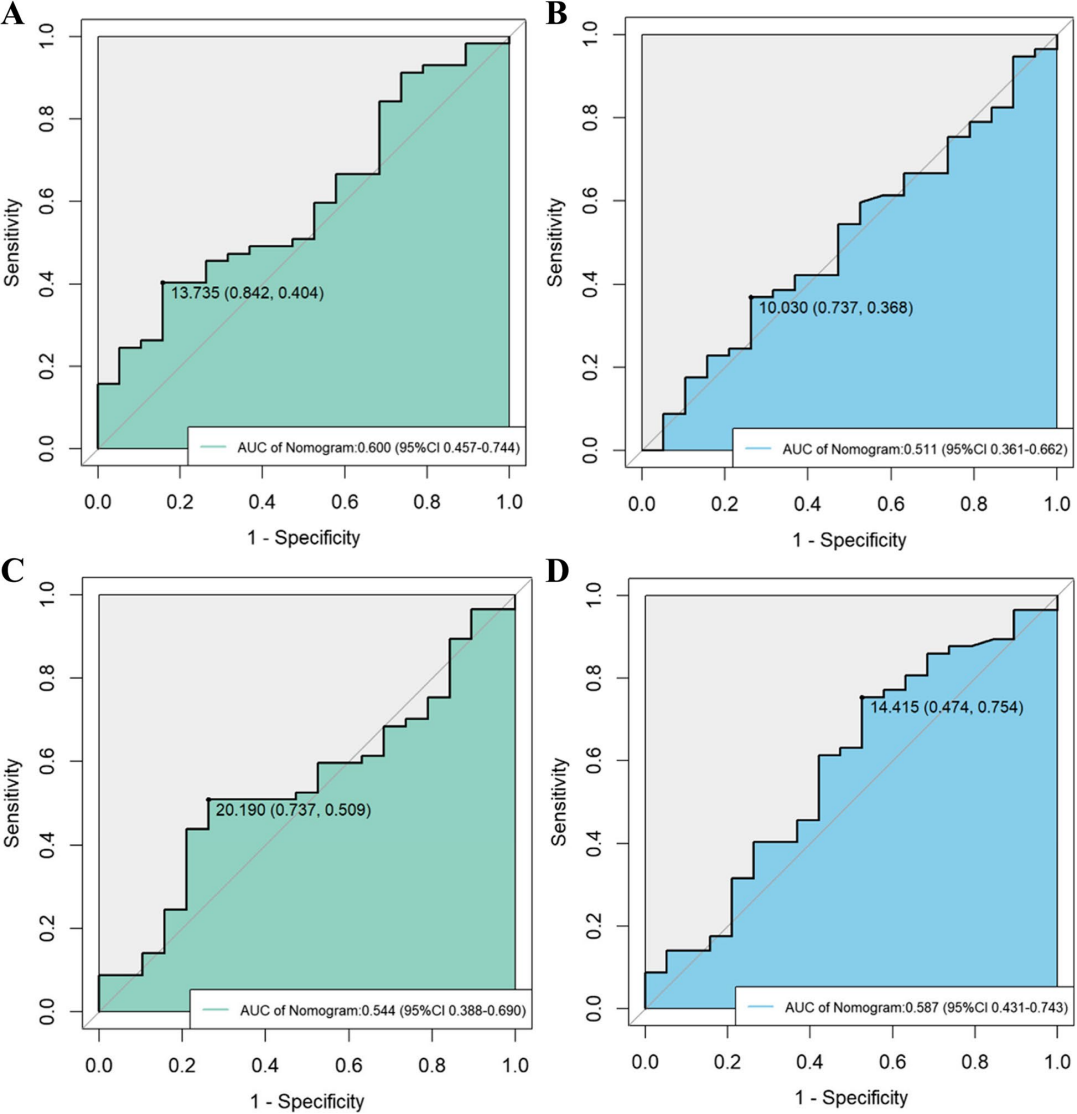
ROC curves for TTP of Arteries in Knee Joint (ROI 2) and Ankle Joint (ROI 3). **A** Preoperative ROI 2 TTP (AUC = 0.600; 95% *CI* 0.457, 0.744; cutoff = 13.735, specificity = 84.20%, sensitivity = 40.40%). **B** Postoperative ROI 2 TTP (AUC = 0.511; 95% *CI* 0.361, 0.662; cutoff = 10.030, specificity = 73.70%, sensitivity = 36.80%). **C** Preoperative ROI 3 TTP (AUC = 0.544; 95% *CI* 0.388, 0.690; cutoff = 20.190, specificity = 73.70%, sensitivity = 50.90%). **D** Postoperative ROI 3 TTP (AUC = 0.587; 95% *CI* 0.431, 0.743; cutoff = 14.415, specificity = 47.70%, sensitivity = 75.40%)

Following the endovascular procedure, no significant difference in TTP was observed for the femoral artery bifurcation ROI (ROI 1) compared to the pre-endovascular procedure (Table 1). A notable decrease in TTP was observed post-endovascular procedure for the knee joint space popliteal artery ROI (ROI 2) and ankle joint space target artery ROI (ROI 3). △TTP2 and △ABI show a significant negative correlation (*r* = − 0.431, *P* < 0.001), and △TTP3 also exhibits a significant negative correlation with △ABI (*r* = − 0.502, *P* < 0.001). ROC curves were constructed for predicting of clinical success based on the pre- and post-endovascular procedure TTP values of ROI 2 and 3, respectively, with corresponding cutoff values obtained (Fig. 3). The AUC values for predicting clinical success using pre- and post-endovascular TTP values in both ROI 2 and 3 were not high. Specifically, pre-endovascular TTP in ROI 2 (AUC = 0.600, 95% *CI* 0.457, 0.744, cutoff value 13.735, sensitivity 40.40%, and specificity 84.20%), post-endovascular TTP in ROI 2 (AUC = 0.511, 95% *CI* 0.361, 0.622, cutoff value 10.030, sensitivity 36.8%, and specificity 73.70%), pre-endovascular TTP in ROI 3 (AUC = 0.544, 95% *CI* 0.388, 0.690, cutoff value 20.190, sensitivity 50.90%, and specificity 73.70%), and post-endovascular TTP in ROI 3 (AUC = 0.587, 95% *CI* 0.431, 0.743, cutoff value 14.415, sensitivity 75.40%, and specificity 47.40%).

### Predictive ability of iFlow parameters for clinical outcomes

After categorizing the TTP of ROI 2 and 3 based on cutoff values, a multivariate logistic regression analysis was conducted to assess the predictive capability of each TTP for clinical success. The results revealed that post-endovascular procedure ankle joint TTP emerged as a risk factor for predicting clinical success (OR 7.21; 95% *CI* 1.68, 35.21; *P* = 0.010). However, the corresponding C-index for this predictor was 0.612, indicating a relatively modest predictive performance (Table 4).

**Table 4:** Adjusted iFlow parameters predicting clinical outcomes using cutoff Values

Parameters	OR (95% *CI*)	*P*	C-statistic
Pre-TTP of ROI 1	1.28 (0.63, 2.76)	0.511	0.571
Pre-TTP of ROI 2	3.76 (0.97, 20.03)	0.078	0.600
Pre-TTP of ROI 3	2.58 (0.57, 11.49)	0.207	0.514
Post-TTP of ROI 1	0.68 (0.35, 1.20)	0.199	0.550
Post-TTP of ROI 2	0.97 (0.24, 3.64)	0.964	0.511
Post-TTP of ROI 3	7.21 (1.68, 35.21)	0.010	**0.612**

ROI 1, above the bifurcation of the common femoral artery; ROI 2, the popliteal artery of the knee joint space; ROI 3, the targeted vessel location within the ankle joint space

## Discussion

PAD exerts significant adverse effects on patients, extending beyond impaired limb functionality to a strong association with heightened mortality rates and increased risk of other cardiovascular diseases such as myocardial infarction and ischemic stroke [13]. The management of PAD involves various approaches, including lifestyle interventions, pharmacological treatments, exercise therapy, and endovascular interventions. When conservative approaches fail to ameliorate patient symptoms, endovascular intervention becomes a necessary means to restore lower limb arterial circulation. Despite significant advancements in endovascular interventions, which have enabled effective lower limb arterial revascularization through techniques like balloon angioplasty and stent placement, patients with PAD frequently present with comorbidities such as diabetes or other systemic conditions. These factors may contribute to variations in individual clinical outcomes even when employing endovascular treatment methods. Based on the research conducted by Korosoglou et al. [14], the outcomes of post-revascularization in PAD patients with diabetes mellitus have yielded less favorable results. Due to the fact that achieving technical success does not always guarantee clinical success, it is necessary to employ additional techniques to aid in predicting clinical outcomes.

Currently, the initial assessment of endovascular procedure efficacy often relies on visual evaluation of DSA images, based on the observation of intravascular contrast agent filling, but this approach lacks quantification. Traditional arterial perfusion assessment methods, such as DUS and CTA, have proven effective in evaluating perfusion status and providing quantitative parameters. In a preoperative assessment of 94 PAD patients, Ombretta et al. [8] found that in the iliac arterial district, DUS accuracy was lower than that of DSA. In the femoro-popliteal and below-the-knee regions, DUS exhibited good consistency with DSA, but obtaining high-quality images and parameters required a skilled operator. CT perfusion imaging has been widely used to provide pre- and postoperative arterial perfusion parameters for PAD patients, aiding in the assessment of PAD conditions and prognosis. However, it comes with drawbacks such as radiation exposure and a large contrast agent dose. To address these issues, perfusion magnetic resonance imaging (MRI) techniques have gained attention. Besides dynamic contrast-enhanced MRI, blood oxygenation level-dependent MRI, arterial spin labeling MRI, and intravoxel incoherent motion MRI do not require contrast agents and involve no radiation exposure [15]. Nevertheless, these assessment techniques often necessitate specialized equipment and environments, increasing patient medical costs, and are unable to provide real-time evaluations during surgical procedures, presenting a challenge that is currently difficult to resolve. The *Syngo* iFlow utilizes manual contrast bolus chasing angiography, enabling real-time analysis during vascular revascularization. It can be repeated in cases of controversial treatment results. Moreover, it does not require additional contrast agents or radiation exposure. Several researchers have published studies on the use of iFlow to evaluate post- interventional effectiveness of endovascular treatments, providing insights into the improvements of lower limb circulation [9, 16, 17]. The *Syngo* iFlow technology comprises two core functionalities: 1) converting DSA sequences into color-coded images for visual assessment by operators, and 2) enabling quantitative assessment of selected ROIs based on variations in intravascular contrast concentration [18]. In our study, we observed that the visual scores based on iFlow showed slightly higher sensitivity, specificity, positive predictive value, negative predictive value, and accuracy compared to the use of DSA images by the two physicians. However, the difference between the two approaches was not significant. Simultaneously, the consistency of physicians in visual assessment using both DSA and iFlow images was moderate and displayed no significant disparity. Despite enhancing the contrast between arteries and surrounding tissues, iFlow’s color-coded images are essentially generated by transforming DSA images. Consequently, although the two evaluation methods differ in image presentation, these distinctions do not significantly impact the overall performance of the assessment. It is noteworthy that iFlow images can display the filling status of all intravascular contrast agents, allowing operators to instantly obtain the filling status of all arterial vessels within the image. This eliminates the need to await changes in image frames and enables operators to assess vascular perfusion rapidly and comprehensively, providing expedient information for decision-making.

This study also examined the effectiveness of quantitative assessment using iFlow. The results demonstrated a significant reduction in postoperative TTP for arteries in both the knee joint space and ankle joint space. There is a noticeable correlation between the changes in TTP (△TTP) and the Ankle-Brachial Index (△ABI) for both locations, consistent with the findings of the study conducted by Li et al. [9] To further investigate the predictive value of the aforementioned four parameters on clinical outcomes, their respective cutoff values were obtained and adjusted. Subsequently, through multivariate logistic regression analysis, postoperative ankle joint TTP was identified as a significant risk factor for clinical success (OR 7.21; 95%*CI* 1.68, 35.21), with a C-statistic of 0.612, demonstrating statistical significance in predicting clinical outcomes. Predicting post-endovascular procedure clinical outcomes holds paramount significance in establishing subsequent treatment strategies, particularly for patients with severe limb ischemia. This is crucial as the ultimate goal of the endovascular procedure is to reduce amputation rates or severity [19]. Therefore, gaining early insight into the efficacy of vascular reconstruction through imaging methods becomes particularly crucial. This not only aids in personalized patient management but also prevents unnecessary amputation surgeries or treatment delays. We believe that quantitative analysis of iFlow enables operators to understand post-revascularization perfusion improvements in target arteries. Despite limitations in predicting clinical outcomes, it retains practical utility. Moreover, this imaging modality offers swiftness, safety, and reproducibility, with the utmost significance being its intraoperative accessibility.

This study has several limitations. First, our data source stems from a relatively limited single-center institution, acquired retrospectively, possibly constraining the applicability of study outcomes in broader contexts. Therefore, prospective studies are essential to further validate our findings. Second, compared to conventional DSA imaging, iFlow imaging requires a larger amount of iodinated contrast agent for acquisition, which to some extent imposes limitations on patients with compromised renal function.

## Conclusion

To summarize, *Syngo* iFlow color-encoded images provide operators with a comprehensive visualization of lower limb arterial perfusion. This technique has potential clinical applications in evaluating the technical efficacy of interventions after endovascular procedures, with its quantitative analysis parameters demonstrating promising potential in prognosticating clinical success.

## Data Availability

The datasets used and/or analysed during the current study are available from the corresponding author on reasonable request.

## Abbreviations

PAD: Peripheral artery disease
TTP: Time to peak
ROI: Region of interest
ROI 1: Located above the bifurcation of the common femoral artery.
ROI 2: Positioned within the popliteal artery at the knee joint.
ROI 3: Placed at the targeted vessel location within the ankle joint.
DSA: Digital subtraction angiography
TIC: Time-intensity curve
ABI: Ankle-brachial index
TcPO2: Transcutaneous oxygen partial pressure
PPV: Positive predictive value
NPV: Negative predictive value
DUS: Duplex ultrasound
CTA: Computed tomography angiography
NIRS: Near-infrared spectroscopy
